# Association of multidisciplinary collaborative nursing with cognitive function and quality of life after craniotomy for glioma resection: a retrospective comparative study

**DOI:** 10.3389/fmed.2026.1807037

**Published:** 2026-05-20

**Authors:** Xiao-Ping Zhang, Qian Zhang, Ya Zhang, Wei Geng, Tao Zhou, Xiao-Peng Li, Xiao-Fang Qi

**Affiliations:** 1 Department of Neurosurgery, Handan First Hospital, Handan, Hebei, China; 2 Department of Oncology, Handan First Hospital, Handan, Hebei, China; 3 Health Service and Counseling Center, Handan First Hospital, Handan, Hebei, China

**Keywords:** cognitive function, craniotomy, EORTC QLQ-C30, glioma, Montreal cognitive assessment, multidisciplinary collaborative nursing, postoperative recovery, quality of life

## Abstract

**Background:**

Patients undergoing craniotomy for glioma resection may experience perioperative cognitive fluctuations and reduced postoperative quality of life, highlighting the need for standardized perioperative supportive care.

**Aim:**

To assess the association of multidisciplinary collaborative nursing with cognition and quality of life in patients undergoing craniotomy for glioma resection.

**Methods:**

This single-center retrospective comparative study included 83 patients with glioma who underwent craniotomy and tumor resection. Patients were categorized into a routine care group and a multidisciplinary collaborative nursing group according to the nursing model documented during the study period. Cognitive function was evaluated using the Montreal Cognitive Assessment, whereas quality of life was assessed using the European Organization for Research and Treatment of Cancer Quality of Life Questionnaire-Core 30 at baseline, discharge, and 3 months after surgery. Recovery indicators and postoperative complications were also recorded.

**Results:**

The intervention group showed higher MoCA scores at 3 months, and cognitive responders were more frequent in the intervention group. Global health status showed a similar time-dependent pattern, with higher follow-up scores and greater baseline-to-follow-up improvement in the intervention group, whereas the minimally clinically important difference responder rate did not reach statistical significance. Earlier ambulation and shorter drain or catheter duration were observed in the intervention group. The intervention group also had a lower overall complication rate, and postoperative delirium was less frequent.

**Conclusion:**

Multidisciplinary collaborative nursing was associated with more favorable cognitive recovery and selected postoperative outcomes during early rehabilitation after glioma resection.

## 1 Introduction

Glioma is one of the most common malignancies of the central nervous system, and surgical resection remains a key element of treatment (1). Despite significant progress in surgical techniques and perioperative care, a substantial number of patients have functional impairments that extend beyond their survival outcomes (2). Consequently, cognitive function and health-related quality of life have been established as relevant outcome parameters in neuro-oncology (1).

Cognitive deficits are common among patients with gliomas, and may persist or develop further after surgery and adjuvant treatment (3). Postoperative cognitive deficits can be detected in the very early stages after surgery, and short-term impairment has been observed in clinical populations using standardized neuropsychological assessment methods (4). Postoperative cognitive trajectories after glioma resection may differ among individuals, and a decline may still be detected several months after therapy (5). These findings highlight the need for structured perioperative support strategies toward preservation of cognitive function and to facilitate recovery during the immediate postoperative stage and after discharge (6).

Quality of life is also impaired in patients with glioma, with perturbations in several domains, including physical functioning, fatigue, emotional well-being, and social participation, which affect patient-reported outcomes (1). The European Organization for Research and Treatment of Cancer Quality of Life Questionnaire-Core 30 has been widely used to characterize perioperative and follow-up changes in quality of life among patients with glioma, often showing early postoperative decline with partial recovery over time (7). Postoperative cognitive and patient-reported quality of life changes in those with grade 2 and grade 3 diffuse gliomas have also been shown to transiently evolve, thus advocating for follow-up rather than a one-time postoperative assessment (8). Clinically significant impairments may persist in subgroups of patients when overall quality of life appears to be stable, highlighting the need for an individualized approach to supportive care (9).

The early postoperative period after craniotomy is also marked by complications that can counteract neurological recovery and influence patient experience. Delirium after surgery is a well-established type of acute brain dysfunction that has been linked to prolonged hospitalization and poor functional outcomes in surgical populations (10). Delirium occurs with some frequency during neurosurgery and is associated with patient characteristics, perioperative events, and postoperative physiology (11). These perioperative susceptibilities establish a clinical scenario in which integrated prevention, observation, and early rehabilitation could potentially influence not only cognitive outcomes but also quality of life (12).

Enhanced recovery after surgery pathways facilitate perioperative care delivery and have gained increasing attention in cranial neurosurgery (13). Previous studies have indicated that neurosurgical enhanced recovery pathways may be associated with enhanced functional/symptom outcomes at follow-up for patients with glioma, although application and uptake differ regionally (14). Modern glioma quality-of-life models also identify fatigue, sleep disturbances, anxiety, and neurocognitive deficits as potential modifiable factors that can be addressed through systematic supportive care interventions (2).

In the present study, the proposed care model emphasized coordinated perioperative assessment, cognitive-oriented nursing support, delirium-focused prevention, structured early mobilization, symptom management, and continuity of care after discharge (6, 13). Nevertheless, clinical studies focusing on cognitive recovery and quality-of-life trajectories in glioma resection with predetermined time points based on clinically meaningful responder metrics are warranted (8).

**Figure 1:**
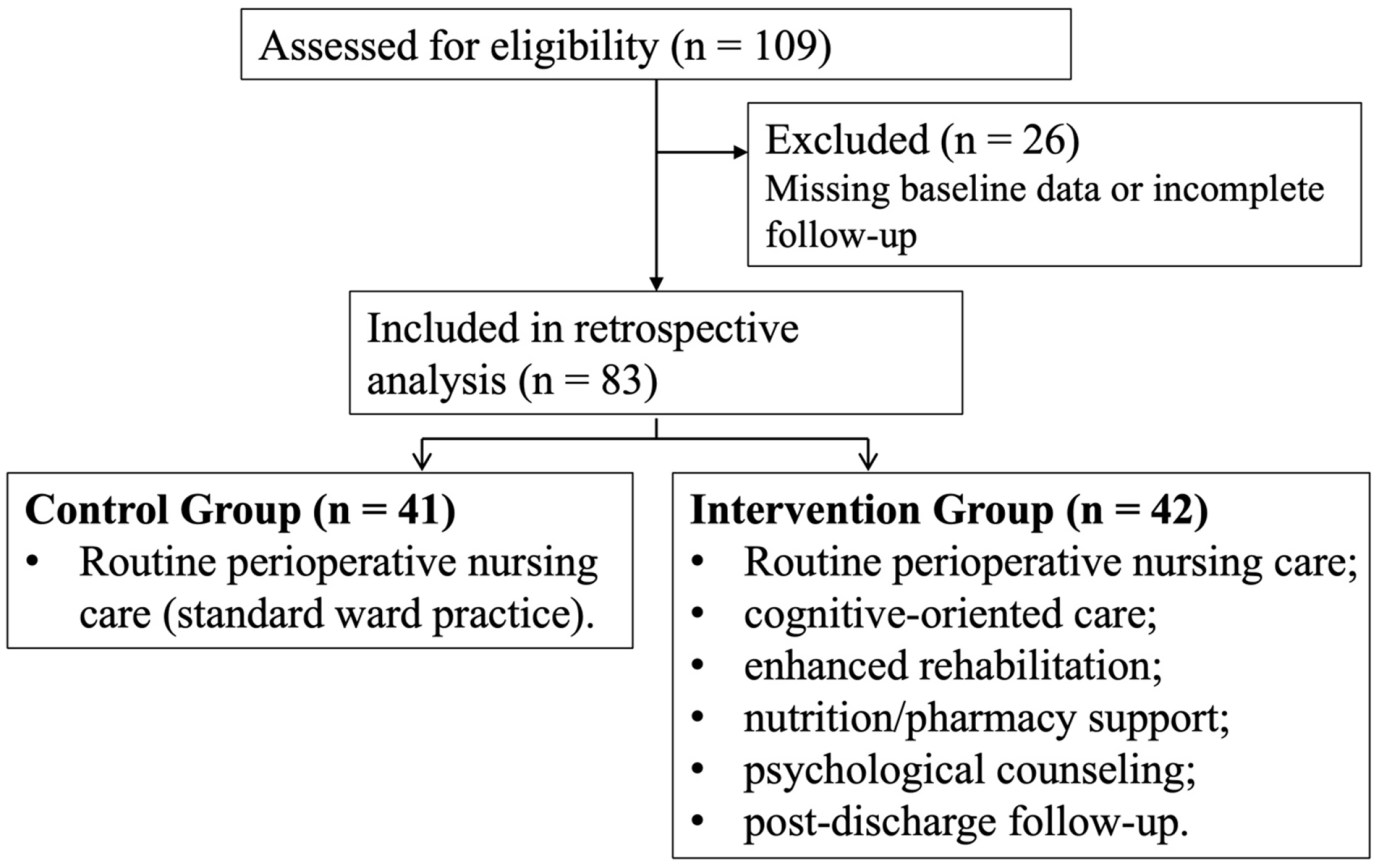
Flow diagram of study enrollment and group assignment.

**Figure 2:**
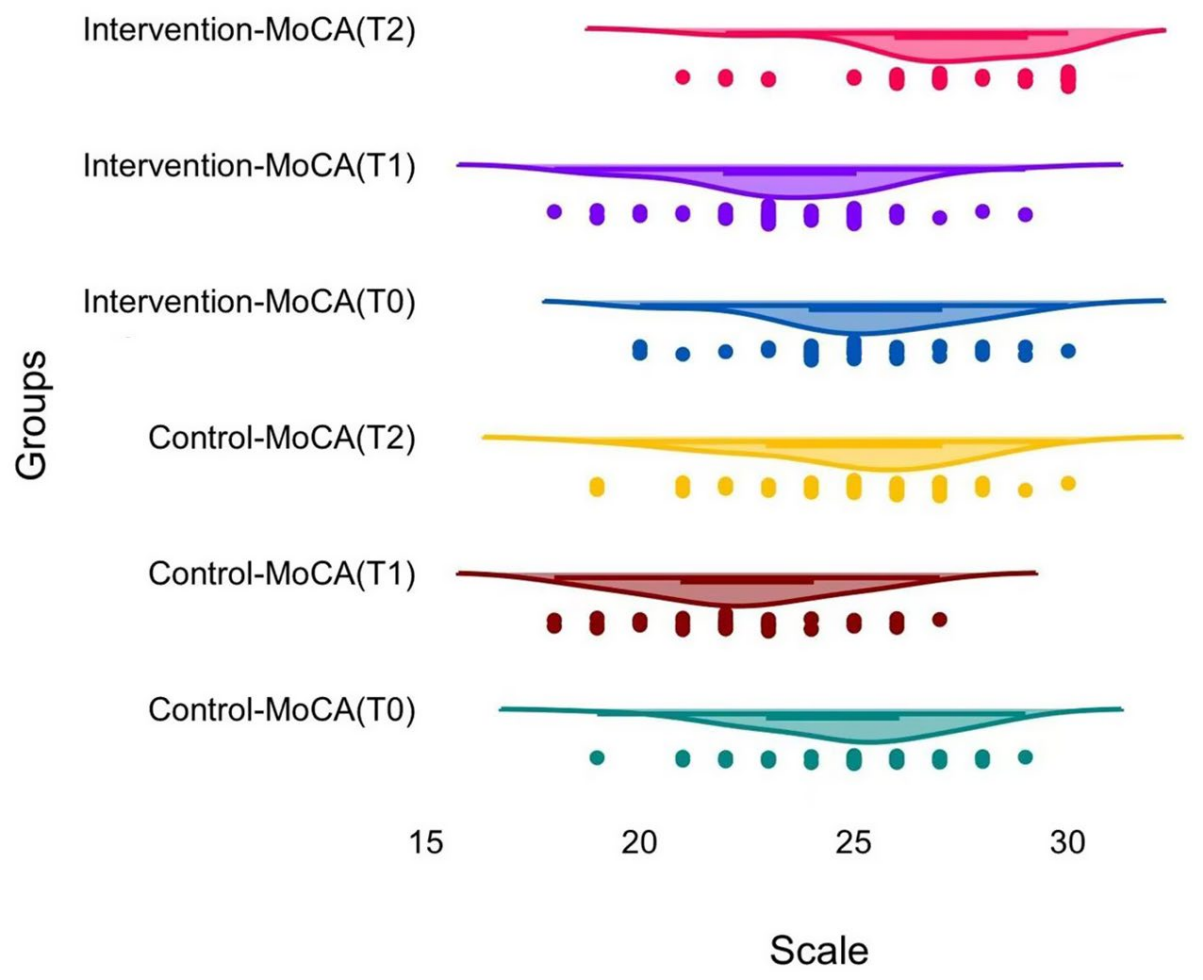
Changes in cognitive function (MoCA scores) over time in the two groups.

**Figure 3:**
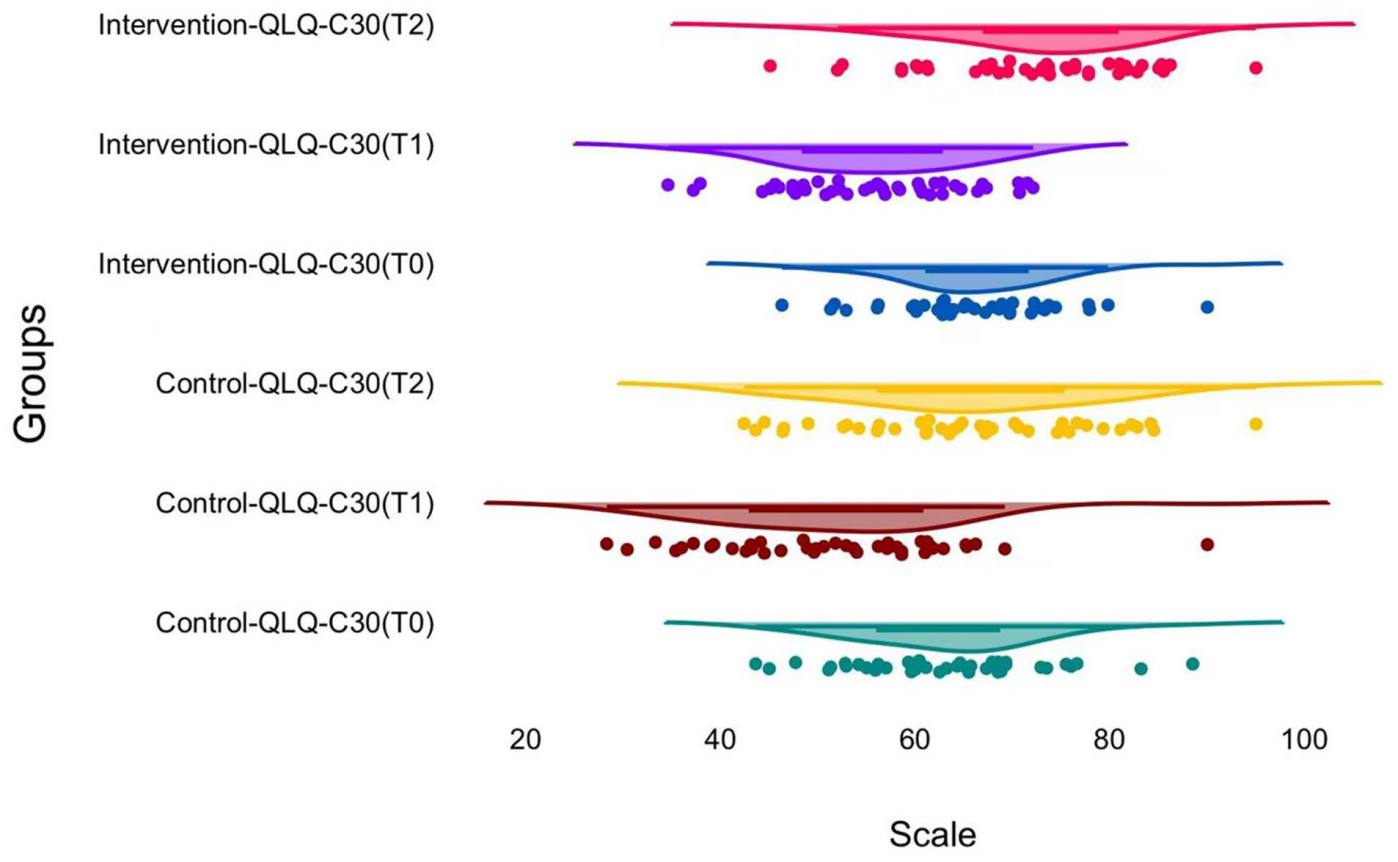
Changes in global quality of life by EORTC QLQ-C30 Global health status over time.

Accordingly, the present study aimed to evaluate the association of a multidisciplinary team-based collaborative nursing model with cognitive status and quality of life in patients with glioma undergoing craniotomy. Cognitive performance was assessed using the Montreal Cognitive Assessment, whereas quality of life was assessed using the EORTC QLQ-C30. Assessments were performed before surgery as baseline, at discharge as the early postoperative time point, and at 3 months after surgery as follow-up (7). In addition to changes in the mean scores of these scales, responder analyses were also applied to enhance clinical interpretability by depicting the number of patients who achieved significant cognitive and clinically meaningful quality-of-life improvements over the recovery period (12) (see Figures 1–3).

## 2 Materials and methods

### 2.1 Study design and setting

This was a single-center retrospective comparative study. Clinical records of patients who underwent craniotomy for glioma resection were reviewed. Patients were categorized into a routine perioperative nursing group and a multidisciplinary collaborative nursing group according to the nursing model documented during the study period. Clinical information and outcome measures were extracted from the electronic medical record system and entered into a de-identified dataset for analysis. This study covered the period from March 7, 2018 to January 17, 2025.

### 2.2 Participants

Individuals with pathologically confirmed glioma who underwent craniotomy during the study period were eligible for inclusion. Patients who completed baseline assessment before surgery and underwent postoperative assessments at discharge and follow-up were included. Patients lacking key outcome measures at any time point, or if follow-up data could not be collected were excluded.

Sample size was determined by the number of eligible consecutive patients available during the study period. Among 109 patients assessed for eligibility, 26 were excluded according to the predefined criteria, and 83 patients were included in the final analysis.

### 2.3 Group assignment

Patients were grouped according to the nursing model recorded during hospitalization. The control group received routine perioperative nursing care based on standard ward practice, whereas the intervention group received the multidisciplinary collaborative nursing program. Because of the retrospective design, no prospective randomization or allocation concealment procedure was applied. Group classification was based on actual care delivery documented in the medical record. The two nursing models were applied during overlapping calendar periods rather than in strictly sequential periods.

### 2.4 Routine nursing care in the control group

The control group received routine perioperative care based on standard clinical practice, which included general education about the disease, perioperative advice, close observation of vital signs and neurological status, medication administration as per protocol, conventional wound care and drainage management, managing pain and sleep, guidance for early mobilization, routine dietary guidance, and discharge advice.

### 2.5 Multidisciplinary collaborative nursing intervention

The intervention group received a multidisciplinary team-based nursing care model, including integrated care planning and individualized recovery assistance, with team members including neurosurgeons, ward nurses, rehabilitation therapists, clinical pharmacists, nutritionists, and mental health counselors. Team members communicated regularly throughout the care period and adapted the care plan as patient condition and recuperation targets changed.

#### 2.5.1 Coordinated assessment and individualized care planning

After admission, structured nursing evaluations were conducted, with an emphasis on functionality, cognitive risks, sleep, diet, activity, and the possibility of postoperative complications. Accordingly, a personalized care plan was developed and revised during the hospital stay.

#### 2.5.2 Cognitive-oriented support and delirium prevention care

Patients were treated with routine reality orientation and supportive communication. The nursing staff offered instructions to sustain attention and daily participation, with an emphasis on reducing environmental stressors in the ward environment. A delirium prevention strategy was implemented by supporting sleep hygiene, adjusting day and night environmental conditions, promoting early mobilization, and appropriately involving families when possible.

#### 2.5.3 Enhanced rehabilitation and early mobilization pathway

A staged mobilization regimen was implemented to facilitate functional recovery. The rehabilitation protocol consisted of progressive activities in bed and ambulation training depending on patient tolerance and neurological status. Safety checks were completed before mobilization, and training targets were modified as a function of daily advancement.

#### 2.5.4 Nutrition and symptom management support

Nutritional status was monitored perioperatively. Dietary advice was provided, and feeding was advanced according to postoperative recovery and tolerance. Symptom-directed treatments were provided to maintain comfort and enable participation in rehabilitation, such as the treatment of pain, nausea, constipation, and sleep disturbance, as clinically indicated and as per attending physician’s orders.

#### 2.5.5 Medication coordination and patient education

A pharmacist-led medication review, including counseling on safe use and adherence, was performed when applicable, focusing on the timing of medication, safety reminders, and education to improve adherence. Patients and their caregivers were also provided with comprehensive health education about perioperative precautions and early warning indicators that merit medical intervention.

#### 2.5.6 Continuity of care after discharge

Support, such as follow-up after discharge, was also provided to reinforce rehabilitation adherence and symptom monitoring. Communication channels were maintained for patient guidance and outcome assessments were performed at planned follow-up time points. The major components of the multidisciplinary collaborative nursing program are summarized in Supplementary Table S1.

### 2.6 Outcome measures and time points

All outcomes were assessed at the following 3 time points: T0 was defined as the baseline time after admission and before surgery during which cognitive function and quality of life assessments were performed; T1 was defined as the early postoperative time point at discharge, with outcome measures recorded on the day of discharge; and T2 was defined as the postoperative follow-up assessment at 3 months after surgery, with an allowable window of plus or minus 2 weeks. Follow-up assessments were completed during outpatient review or telephone follow-up, depending on patient availability. MoCA reassessment at T2 was based on in-person follow-up records, whereas telephone follow-up was used for outcome confirmation and collection of non-cognitive follow-up information when applicable.

The primary outcome was cognitive function, measured using the MoCA. Secondary endpoints included quality of life measured using the EORTC QLQ-C30 global health status scale and physical functioning scale. Postoperative recovery was evaluated according to length of hospital stay, time to first ambulation, time to oral intake, and duration of drainage or catheterization. Complications occurring within 30 days postoperatively were documented during hospitalization and after discharge, if possible.

Postoperative delirium was identified by retrospective review of inpatient medical records. Cases were determined according to documented clinical diagnosis by the treating team during hospitalization. Because of the retrospective design, no independent blinded reassessment was performed.

### 2.7 Responder definitions

Responder definitions were prespecified to enhance clinical interpretability; in this research, a cognitive responder was defined as a patient with an increase of ≥ 2 points on the MoCA at T2 compared with T0. A minimally clinically important difference (MCID) responder for global health status was defined as an increase ≥ 10 points in the QLQ-C30 global health status at T2 relative to T0.

### 2.8 Statistical analysis

Analyses were performed using SPSS version 26.0 (IBM Corp., Armonk, NY, United States). Continuous variables are expressed as mean and standard deviation (SD), and categorical variables are reported as frequency and percentage. Between-group comparisons of continuous outcomes were performed using independent sample *t*-tests. Categorical endpoints were compared using the χ^2^ test or Fisher’s exact test, as appropriate. Changes in scores were calculated as T1 − T0 and T2 − T0, and between-group differences in these changes were evaluated. In addition, linear mixed-effects models with fixed effects for group, time, and group-by-time interaction and a random intercept for participant were used to evaluate longitudinal changes in MoCA and QLQ-C30 outcomes. A two-sided *p* value below 0.05 was considered statistically significant.

## 3 Results

### 3.1 Participant characteristics

Data from 83 patients were analyzed, with 41 and 42 patients in the control and intervention groups, respectively (Table 1). The available baseline variables, including age and sex, did not differ significantly between groups, with mean (± SD) ages of 54.12 ± 15.11 and 50.74 ± 18.45 years in the control and intervention groups, respectively (*p* = 0.363). The sex ratio was also essentially identical, with 21 males and 20 females in the control group, and 21 males and 21 females in the intervention group (*p* = 1.000).

**Table 1:** Baseline characteristics of the included patients.

Variable	Control group (*N* = 41)	Intervention group (*N* = 42)	*p* value
Age (years)	54.12 ± 15.11	50.74 ± 18.45	0.363
Sex (male/female)	21/20	21/21	1.000

T0 = preoperative baseline; T1 = discharge day; T2 = 3-month follow-up.

### 3.2 Cognitive outcomes

Cognitive function exhibited a perioperative pattern in both groups, decreasing at discharge and improving at the three-month follow-up. There was no statistical difference in baseline MoCA scores between the control (24.88 ± 2.23) and intervention (25.19 ± 2.43) groups.

Both groups exhibited decreased MoCA scores from baseline at discharge. At this early postoperative time point, the intervention group had a better score (23.38 ± 2.46) than that in the control group (22.34 ± 2.28) (*p* = 0.049). Cognition scores improved in both groups at 3 months after surgery, although the separation between groups was greater (at T2, the intervention group scored 27.12 ± 2.38 while the control group scores 24.93 ± 2.62) (*p* < 0.001).

In addition, differences between baseline and follow-up were evaluated. As shown in Table 2, the mean change from T0 to T2 in MoCA was 1.93 ± 1.22 in the intervention group and 0.05 ± 1.34 in the control group (*p* < 0.001). Furthermore, according to the responder criterion, the responder rates were 66.7% in the intervention group and 14.6% in the control group (*p* < 0.001). Longitudinal analysis using a linear mixed-effects model also showed a significant group-by-time interaction for MoCA at T2, with an estimated interaction effect of 1.88 points (95% CI: 1.34 to 2.42; p < 0.001), supporting differential cognitive recovery trajectories between groups (see Tables 3–5).

**Table 2:** Cognitive outcomes (MoCA) at T0, T1, and T2.

Outcome	Control group	Intervention group	*p* value
MoCA at T0	24.88 ± 2.23	25.19 ± 2.43	0.543
MoCA at T1	22.34 ± 2.28	23.38 ± 2.46	0.049
MoCA at T2	24.93 ± 2.62	27.12 ± 2.38	< 0.001
Change in MoCA (T1 − T0)	−2.54 ± 0.84	−1.81 ± 0.77	< 0.001
Change in MoCA (T2 − T0)	0.05 ± 1.34	1.93 ± 1.22	< 0.001
Cognitive responder (MoCA increase ≥2 at T2 vs. T0), *n* (%)	6 (14.6%)	28 (66.7%)	< 0.001

T0 = preoperative baseline; T1 = discharge day; T2 = 3-month follow-up. MoCA range 0–30; higher scores indicate better cognition.

**Table 3:** Quality of life outcomes by EORTC QLQ-C30.

Outcome	Control group	Intervention group	*p* value
QLQ-C30 global health status at T0	63.4 ± 9.8	65.9 ± 8.4	0.219
QLQ-C30 global health status at T1	51.6 ± 12.3	55.7 ± 9.5	0.094
QLQ-C30 Global health status at T2	65.5 ± 12.7	72.3 ± 10.5	0.009
Change in global health status (T1 − T0)	−11.9 ± 6.6	−10.3 ± 6.4	0.262
Change in global health status (T2 − T0)	2.0 ± 7.6	6.4 ± 7.0	0.007
QLQ-C30 physical functioning at T0	75.3 ± 12.2	74.0 ± 13.3	0.638
QLQ-C30 physical functioning at T1	58.0 ± 16.2	60.3 ± 16.3	0.504
QLQ-C30 physical functioning at T2	78.6 ± 14.5	80.3 ± 13.3	0.585
MCID responder for Global health status (≥10-point increase at T2 vs. T0), *n* (%)	6 (14.6%)	13 (31.0%)	0.132

T0 = preoperative baseline; T1 = discharge day; T2 = 3-month follow-up. QLQ-C30 global health status and physical functioning range 0–100; higher scores indicate better QoL/functioning.

**Table 4:** Postoperative recovery indicators.

Indicator	Control group	Intervention group	*p* value
Length of stay (days)	10.80 ± 4.01	10.17 ± 3.11	0.421
Time to first ambulation (days)	3.02 ± 0.99	2.01 ± 0.78	<0.001
Time to oral intake (days)	2.98 ± 1.32	2.78 ± 1.14	0.469
Drain/catheter duration (days)	4.84 ± 1.86	3.87 ± 1.66	0.015

**Table 5:** Postoperative complications.

Complication	Control group	Intervention group	*p* value
Any complication, *n* (%)	18 (43.9%)	8 (19.0%)	0.028
Pulmonary infection, *n* (%)	4 (9.8%)	1 (2.4%)	0.202
Intracranial infection, *n* (%)	2 (4.9%)	2 (4.8%)	1.000
Postoperative delirium, *n* (%)	8 (19.5%)	0 (0.0%)	0.002
Deep vein thrombosis, *n* (%)	1 (2.4%)	1 (2.4%)	1.000
Pressure injury, *n* (%)	1 (2.4%)	1 (2.4%)	1.000
30-day readmission, *n* (%)	2 (4.9%)	3 (7.1%)	0.616
Other, *n* (%)	0 (0.0%)	0 (0.0%)	1.000

### 3.3 Quality of life outcomes

Analysis of global quality of life revealed a similar time-dependent trend, with the lowest scores at discharge and better scores at the three-month follow-up. At baseline, global health status was 63.4 ± 9.8 and 65.9 ± 8.4 in the control and intervention groups, respectively; at discharge, these values dropped to 51.6 ± 12.3 and 55.7 ± 9.5, and the between-group difference was non-significant at this point (*p* = 0.094).

Global health status improved in the intervention group at the three-month follow-up. The mean scores were 72.3 ± 10.5 and 65.5 ± 12.7 for the intervention and control groups, respectively (*p* = 0.009). Similarly, the change from baseline to follow-up was greater in the intervention group. Changes from T2 to T0 were 6.4 ± 7.0 and 2.0 ± 7.6 for the intervention and control groups, respectively (*p* = 0.007).

Physical performance scores also decreased at discharge and increased at T2; however, no significant between-group differences were found. At 3 months, the physical functioning score in the intervention group was 80.3 ± 13.3 and, in the control group, 78.6 ± 14.5 (*p* = 0.585). The MCID responder status for global health status was assessed using the criterion of improvement of ≥ 10 points at T2 in relation to T0. The response rate was higher in the intervention group (31.0%) than that in the control group (14.6%), although the difference was not statistically significant (*p* = 0.132).

A significant group-by-time interaction was also observed for global health status at T2 in the linear mixed-effects model, with an estimated interaction effect of 4.40 points (95% CI: 1.05 to 7.76; *p* = 0.010), whereas no significant interaction was observed for physical functioning (interaction estimate 3.00 points, 95% CI: −1.18 to 7.18; *p* = 0.160).

### 3.4 Postoperative recovery indicators

Several early postoperative recovery milestones exhibited significant between-group differences. Time to first ambulation was shorter in the intervention group (2.01 ± 0.78 days) compared with 3.02 ± 0.99 days in the control group (*p* < 0.001). Drainage time or catheter time was also significantly shorter in the intervention group (3.87 ± 1.66 days) than that in the control group (4.84 ± 1.86 days) (*p* = 0.015). However, there were no significant differences in length of hospital stay or time to oral intake between the two groups.

### 3.5 Postoperative complications

The incidence of postoperative complications differed between the groups. Complications of any type were reported in 19.0% of patients in the intervention group and 43.9% of patients in the control group, with a *p* value of 0.028. Among the recorded complications, postoperative delirium showed the largest between-group difference in this cohort, although this finding should be interpreted cautiously because delirium was identified from retrospective chart documentation rather than standardized prospective screening. Other complications, such as pulmonary infection, intracranial infection, deep vein thrombosis, pressure injury, and 30-day readmission, did not differ significantly between the groups in this cohort.

## 4 Discussion

MoCA scores exhibited a postoperative decline at discharge, followed by recovery at 3 months, which is consistent with the perioperative cognitive fluctuation described in neurosurgical and neuro-oncology cohorts (15). In glioma populations, cognitive function at approximately 3 months after surgery has been repeatedly assessed as a clinically meaningful stage of recovery and stabilization; however, inter-individual variability remains substantial (16). This time-dependent pattern provides an appropriate background for interpreting the group differences observed at discharge and during follow-up (15).

In the present study, the intervention group exhibited a less pronounced early postoperative decline and a more favorable level of cognitive recovery by 3 months. These findings are consistent with the concept that cognitive outcomes after brain tumor surgery are influenced not only by surgical factors, but also by perioperative physiological stress, sleep disturbance, mobility restriction, symptom burden, and quality of supportive care (10). Postoperative cognitive impairment has been discussed as a multifactorial phenomenon in surgical populations, and supportive strategies that address modifiable perioperative stressors may be relevant in practical care (17).

Responder analysis was used to improve interpretability at the individual patient level. The higher proportion of cognitive responders in the intervention group indicates that improvement was not restricted to small mean shifts alone but was observed in a larger proportion of patients who met the pre-specified threshold. This approach may be useful in glioma care, where cognitive trajectories are heterogeneous and a single average value can conceal clinically important patterns (15). Similar work has emphasized the clinical relevance of cognitive status during the postoperative and early adjuvant phases, reinforcing the value of using time-linked assessments and patient-level interpretation(s) (16).

Global quality of life also exhibited a decline at discharge with recovery by 3 months, which parallels prospective observations in patients with glioma using the EORTC QLQ-C30 and brain tumor modules during postoperative follow-up (7). In the current study, global health status at 3 months was higher in the intervention group and the improvement from baseline to follow-up was greater. This is consistent with the concept that structured perioperative support can contribute to an improved recovery experience, as captured by patient-reported outcomes (7). Because quality of life reflects multiple interacting domains, including fatigue, sleep disturbance, and emotional burden, it is also plausible that integrated supportive care may influence the global health status domain through several concurrent mechanisms rather than through a single pathway (18). Taken together, the quality-of-life findings appeared mixed, with clearer between-group separation in global health status than in physical functioning, while the MCID responder comparison did not reach statistical significance.

The MCID-based responder analysis for global health status revealed a higher proportion of responders in the intervention group, although the difference was not statistically significant. This pattern can occur when improvement is present, but does not consistently exceed a relatively stringent responder threshold in a modest-size sample. Analogous issues have been raised in neuro-oncology quality-of-life studies (6), wherein there may be clinically meaningful fluctuations in subsets, whereas comparisons at the group level remain vulnerable to sample size and variance, which may reflect variability in individual-level improvement and limited statistical power for responder-based comparisons (7). The modest divergence in physical functioning in the current study may also be due to neurological status, compliance with rehabilitation, and adjuvant treatment protocols, each of which may influence functional outcomes that vary over time (18).

The intervention group exhibited significantly earlier ambulation and shorter drain or catheter periods, which is consistent with perioperative recovery pathways recommending early ambulation (19). Real-world glioma resection populations have demonstrated that early mobilization protocols may facilitate early recovery without causing harm, indicating that they are practical in neurosurgical practice (19). Early indicators of recovery have also been shown to improve with enhanced recovery paradigms in craniotomies, indicating that perioperative methodologies may affect milestone-based endpoints (20). In addition, enhanced recovery after surgery-based management of gliomas has been reported in real-world implementation studies, reinforcing the relevance of structured perioperative optimization beyond conventional ward practices (21).

The overall complication rate was lower in the intervention group, with postoperative delirium exhibiting the greatest separation between the groups. Delirium after intracranial surgery has been reported with significant frequency and is related to negative outcomes, rendering it a clinically relevant perioperative event for surveillance and prevention (11). Reviews and cohort studies have identified several risk factors for delirium in patients undergoing neurosurgery, including perioperative physiological instability, pain, sleep deprivation, and immobilization (22). Delirium prevention often relies on multicomponent strategies, such as sleep hygiene support, early mobilization, and optimization of the ward environment. These components are operationally feasible within coordinated nursing-led care models and may be relevant in routine clinical practice (10). Additionally, current discussions stress that surveillance and prevention approaches must be practical and neurosurgery specific (23).

The present findings are consistent with emerging evidence supporting integrated care models for patients with gliomas that combine multidisciplinary nursing coordination with enhanced recovery principles (24). Such integrated approaches have reported improvements in recovery indicators and patient-reported outcomes, which support the plausibility of the observed direction of the effect on cognition and quality of life (24). Similarly, for post-glioma surgery rehabilitation needs, it has been demonstrated that information, caregiving, and self-esteem-related needs can influence quality of life at follow-up, which may imply that continuity of care and structured support can be meaningful to intervention programs (6).

Despite these results, several limitations must be considered when interpreting our findings. First, this was a single-center retrospective study with a modest sample size, and because of the retrospective design and the absence of independent blinded reassessment for postoperative delirium, the findings should be interpreted as associations rather than definitive causal effects. The follow-up was only 3 months, which is probably not representative of long-term oncological treatment. Additionally, important tumor- and treatment-related baseline variables, including tumor grade, tumor location, extent of resection, and postoperative adjuvant therapy, were not fully available in the dataset and therefore could not be incorporated into the present analysis. Meanwhile, because the research was centered on patient-reported outcomes, a more detailed assessment using brain tumor-specific modules may potentially uncover more in subsequent studies (7).

## 5 Conclusion

In this retrospective comparative study, multidisciplinary collaborative nursing was associated with more favorable cognitive recovery, higher follow-up global health status, earlier ambulation, shorter drainage or catheter duration, and a lower overall complication burden during early postoperative rehabilitation after glioma resection. Further prospective studies with larger samples and more detailed tumor-related data are needed to validate these findings.

## Data Availability

The original contributions presented in the study are included in the article/Supplementary material, further inquiries can be directed to the corresponding author.
