# Supplementary material for: Association of multidisciplinary collaborative nursing with cognitive function and quality of life after craniotomy for glioma resection: a retrospective comparative study
Source: Front Med (Lausanne). 2026 May 20;13:1807037. doi: 10.3389/fmed.2026.1807037 (PMC13230025; doi:10.3389/fmed.2026.1807037)
Supplement: Supplementary file 1 [file Table_1.DOCX]

**Supplementary Materials**

**Table S1.** Structured summary of the multidisciplinary collaborative nursing program, including personnel involved, timing, frequency, and implementation status.

| **Component** | **Personnel involved** | **Timing** | **Frequency** | **Mandatory or as indicated** |
| --- | --- | --- | --- | --- |
| Coordinated assessment and individualized care planning | Ward nurses, neurosurgeons, rehabilitation therapists | After admission and during hospitalization | At admission, then updated as needed | Mandatory |
| Cognitive-oriented support | Ward nurses | During hospitalization | Daily | Mandatory |
| Delirium prevention care | Ward nurses, family when available | During hospitalization | Daily | Mandatory |
| Early mobilization pathway | Rehabilitation therapists, ward nurses | Postoperative period | Daily, progressed according to tolerance | Mandatory |
| Nutrition support | Nutritionists, ward nurses | Perioperative period | As assessed during hospitalization | As indicated |
| Symptom management | Ward nurses, treating physicians | Perioperative period | As symptoms occurred | As indicated |
| Pharmacist-led medication review | Clinical pharmacists | During hospitalization | When medication adjustment or counseling was needed | As indicated |
| Patient and caregiver education | Ward nurses, pharmacists | During hospitalization and at discharge | Repeated education as needed | Mandatory |
| Post-discharge follow-up | Ward nurses or designated staff | After discharge to 3-month follow-up | At scheduled follow-up contact | Mandatory |
